# Analysis of the Digestion Dynamics and Dietary Risk Assessment of Fluridone in Cotton Fields via QuEChERS Coupled with HPLC

**DOI:** 10.3390/toxics13070526

**Published:** 2025-06-23

**Authors:** Sen Wang, Ruitong Yang, Yuxuan Li, Zhiqiang Jin, Yutian Xia, Yipin Zhao, Xiaoqiang Han, Guoqiang Zhang, Chunjuan Wang, Ting Ma, Cailan Wu, Desong Yang

**Affiliations:** Key Laboratory of Oasis Agricultural Disease and Pest Management and Plant Protection Resource Utilization in Xinjiang, School of Agriculture, Shihezi University, Shihezi 832000, China; wangsen@stu.shzu.edu.cn (S.W.);

**Keywords:** fluridone, QuEChERS, HPLC, cotton field, pesticide digestion

## Abstract

Fluridone is a pyrrolidone soil-sealing herbicide that has been widely used in cotton fields in Xinjiang in recent years. The purpose of this study was to establish a method for determining fluridone residues in cotton fields and to perform residue digestion tests, final residue analysis, and dietary risk assessment. Samples were extracted with acetonitrile, purified with primary secondary amine (PSA) and multi-walled carbon nanotubes (MWCNTs), and analyzed by high-performance liquid chromatography (HPLC). The results showed that in a certain concentration range, the concentration and peak area of fluridone showed a good linear relationship (R^2^ > 0.99), with limit of detection (LOD) and limit of quantification (LOQ) values of 0.00090–0.00108 mg·kg^−1^ and 0.0030–0.0033 mg·kg^−1^, respectively. The relative standard deviation (RSD) values of fluridone were 0.46% to 4.57% at the spiked level of 0.1, 0.5, and 1.0 mg·kg^−1^, respectively. The average daily recovery rate of fluridone was 85.08% to 95.07%. The residual levels of fluridone in cottonseed oil were below the safety threshold, indicating no significant dietary risk to consumers.

## 1. Introduction

Cotton (*Gossypium hirsutum* L.) is an annual herbaceous or perennial shrub plant of the genus Mallow. Cotton is also one of China’s most important cash crops, and its output ranks first globally [1]. Owing to its unique geographical and natural conditions, Xinjiang has emerged as a significant high-quality cotton cultivation region in China [2]. In 2022, the cotton planting area in Xinjiang amounted to approximately 2,496,900 hectares. However, weeds pose a significant threat to cotton yield and quality improvement, as they compete for sunlight, water, fertilizer, and space with cotton plants. Additionally, weeds serve as intermediate hosts for pest and disease transmission, substantially increasing cotton planting costs (fertilizer and pesticide inputs) and irrigation expenses. Consequently, cotton yield and fiber quality decline [3]. The perennial yield loss attributable to weeds ranges from 12.5% to 14.8% [4]. Fluridone, whose chemical name is 1-methyl-3-phenyl-5- (3-trifluoromethylphenyl)-4 (1 H)-pyridone, is a pyrrolidone soil-sealing herbicide widely used in cotton fields in Xinjiang in recent years. It inhibits photosynthesis and causes plant death. It is often paired with pendimethalin, which can control annual broad-leaved and grassy weeds such as nightshade, gray quinoa, and barnyard grass in cotton fields. Pendimethalin has an effective period of about 60 days and is safe for cotton seedling emergence and growth [5,6]. Fluridone has a long residual period in the soil, which poses a certain potential risk of pesticide damage to crops during subsequent rotations [7].

Pretreatment techniques for pesticide residues include methods like QuEChERS (Quick, Easy, Cheap, Effective, Rugged, and Safe), solid phase extraction (SPE), gel permeation chromatography (GPC), and accelerated solvent extraction (ASE) [8,9]. QuEChERS was introduced at the Fourth European Pesticide Residue Group Conference in 2002. This acronym stands for Quick, Easy, Cheap, Effective, Rugged, and Safe. QuEChERS is widely applied for the detection of multiple pesticide residues by removing impurities through interactions between adsorbents and matrix impurities [10]. Compared to traditional methods, QuEChERS is widely applied to detect multiple pesticide residues by removing impurities through interactions between adsorbents and matrix impurities. Compared to traditional methods, QuEChERS offers high recovery rates, often exceeding 85% for numerous polar and volatile pesticides. High precision and accuracy are corrected using the internal standard method, and the approach has the advantages of fast analysis and simple operation, low solvent consumption, minimal environmental impact, cost-effectiveness, and simplicity [11].

China is a major producer of pesticides and uses them extensively. Detecting pesticide residues in soil is crucial for environmental monitoring. Xu Yihong et al. utilized an improved QuEChERS method to detect toxaphene in soil, exploring different polarity extraction solvents and the influence of graphitized carbon black and PSA on purification and recovery rates. This method boasts good recovery, precision, simplicity, and speed [12]. Cui Jun et al. applied an enhanced QuEChERS method to soil sample pretreatment, simplifying and optimizing the steps required. The direct instrument analysis of the extract allowed for the rapid detection of four nitrophenols in soil [13]. Zhang Hongchao et al. employed the QuEChERS method and UPLC-MS/MS to determine the dissipation dynamics and metabolic patterns of six common pesticides in cucumbers, aiming to provide a scientific basis for their use and guide practical production [14].

However, there are few reports on the residual analysis of fluridone at home and abroad. In this study, the optimized QuEChERS pretreatment method was selected and combined with HPLC to establish the residual digestion dynamics and final residue levels of fluridone in the cotton field environment, and a risk assessment was carried out. This study provides a scientific basis for the application of fluridone in the process of weeding in cotton fields, provides a basis for the study of subsequent soil environmental behavior, and provides a reference for the development of analytical methods for fluridone residues in other crops and organisms.

## 2. Materials and Methods

### 2.1. Residue Digestion in the Field and Final Residue Testing

The field experiments were carried out in Shihezi City, Xinjiang, China (44.35° N, 86.01° E, altitude 321.4 m), on April 2023 and April 2024. Shihezi City has a temperate continental climate. In accordance with the Guidelines for Crop Residue, the trial followed the Guidelines for Crop Residue Testing (NY/T 788-2018) issued by the Ministry of Agriculture and Rural Affairs of China and the Standard Operating Procedures for On-site Testing of Pesticide Residues (PVCI, 2021). The cotton cultivar Huiyuan 720 was planted in a randomized complete block design with four replicates. Each plot measured 12 m × 14 m (168 m^2^), separated by 1.5 m buffer zones to minimize drift interference. Fluridone (42% suspension concentrate) was applied pre-emergence at three concentrations: 220.5, 441.0, and 661.5 g a.i.·ha^−1^, using a calibrated backpack sprayer delivering 30 L·ha^−1^. Adjacent control plots received water with equivalent adjuvant (0.1% Tween 20). The detailed physicochemical properties of the soil are shown in Table S1.

Residual dissolution test: Soil samples were collected at 0.083, 1, 3, 7, 14, 21, and 28 days using the five-point sampling method. Subsequent separation was conducted according to the four-point method. Each soil sample, weighing no less than 1 kg, was securely bandaged in a dedicated container and labeled appropriately. The sampling points were evenly distributed to guarantee the representativeness of the collected samples. These samples were stored in a refrigerator at −20 °C in darkened cryovials to detect fluridone residues.

Final residue test: During cotton harvest (September 2023/2024), triplicate samples of soil (0–15 cm depth), cotton leaves, and seeds were collected from randomized 10 × 10 m grids using stratified random sampling (NY/T 788-2018). Plant tissues were lyophilized and ground to a particle size of 100 um.

### 2.2. Collection and Preparation of Samples

Approximately 1 kg of surface soil was randomly collected from multiple points within a depth range of 0 to 10 cm. Post fragment removal allowed the soil to dry naturally. Subsequently, it was ground through a 20-mesh sieve (0.9 mm aperture). Around 1.0 kg of cotton plants were gathered from various locations, chopped, and blended. Additionally, cottonseeds were collected from multiple points and were harvested after harvesting the cotton plants. After drying at 80 °C, the samples were stored at −20 °C until further analysis.

### 2.3. Sample Preprocessing

An optimized QuEChERS method was used for preprocessing the field soil, cotton plants, cottonseeds, and blank matrix samples [15,16]. Once soil collection was complete, the samples were air-dried and ground through a 2 mm sieve for subsequent use. Then, 10.0 g of soil samples, 5.0 g of cotton plant samples, and 5.0 g of cottonseed samples were precisely weighed and placed into 50 mL centrifuge tubes using an electronic balance. Then, 10 mL of acetonitrile was added and extracted for 5 min. Following this, 2.0 g of NaCl was incorporated to facilitate the separation of the aqueous and organic phases. The samples were continuously vortex-mixed for 2 min and then centrifuged at 4000 rpm/min for 5 min to purify the samples. All procedures were repeated three times.

### 2.4. Instrument Test Condition

The chromatographic settings included an injection volume of 2.0 μL, with a mobile phase consisting of acetonitrile and 0.1% formic acid aqueous solution in a 80:20 volume ratio. The flow rate was maintained at 0.3 mL/min, and the column oven temperature was set to 40 °C. The column used was a Sunfire C18-ODS stainless steel column with dimensions of 150 mm × 4.6 mm. The detection wavelength was set at 310 nm.

### 2.5. Preparation of Standard Solutions and Matrix-Matched Standard Solutions

#### 2.5.1. Preparation of Standard Solutions

The fluridone standard (10.0 mg) was precisely measured and dissolved in acetonitrile using an ultrasonic cleaner until a final volume of 10 mL was reached, yielding a stock solution with a concentration of 1000 mg/L. The stock solution was stored at −20 °C. The stock solution was sequentially diluted with acetonitrile to obtain working solutions with concentrations of 10, 1, 0.5, 0.1, and 0.01 mg/L.

#### 2.5.2. Preparation of Matrix-Matched Standard Solutions

Blank samples of the field soil, cotton plants, and cottonseeds were obtained. These samples were pretreated to extract blank matrix solutions. The blank matrix solutions were used to prepare matrix-matched standard solutions with concentration gradients of 10, 1, 0.5, 0.1, and 0.01 mg/L.

#### 2.5.3. Recovery Rate Experiment with Spiking

Recovery rate experiments were conducted to ascertain the accuracy and precision of the methodology. These experiments involved spiking three distinct concentrations of fluridone standards into blank matrices (comprising field soil, cotton plants, and cottonseeds) devoid of fluridone. Each concentration level was subjected to five parallel treatments. Measurements were then performed in accordance with the methodology established within this study. Subsequently, the average recovery rates and relative standard deviations (RSDs) for each concentration level were computed, as detailed in Table 1.

### 2.6. Validation of Analytical Method

The quantitative analytical method was rigorously validated in compliance with the SANTE guidelines. During this validation process, the analytical method’s key parameters were established, including linearity, LOQ, LOD, matrix effect (ME), precision, and accuracy [17,18].

HPLC analysis was conducted on standard solutions, including matrix-matched standards, with five replicates for each concentration. By plotting the mass concentration of fluridone as the *x*-axis and the corresponding peak area as the *y*-axis, a matrix-matched standard curve and correlation coefficient (R) for fluridone were obtained. This evaluation assessed the linear relationship between fluridone concentration and peak area. An R^2^ value greater than 0.99 indicates accurate analysis.

The sensitivity of the analytical method was assessed using the limit of detection (LOD) and LOQ. To determine sensitivity, the lowest concentration was added to selected matrices to meet method specifications. The LOD was defined as the fluridone concentration in the matrix where the signal-to-noise ratio (S/N) reached 3, considering the background noise from the blank matrix. Analogously, the LOQ corresponded to the fluridone concentration with an S/N of 10 [17,19,20].

To assess the matrix effect, a comparison was made between the slope ratio of the solvent standard curve and the matrix-matched standard curve. The formula below was utilized to calculate the matrix effect of the fluridone standard across various matrices:

(1) ME=(B−A)/A×100%

In the formula, “ME” represents the matrix effect; “A” represents the slope of the solvent (acetonitrile) standard curve; and “B” represents the slope of the matrix-matched standard curve (field soil, cotton plants, and cottonseeds).

To ascertain the accuracy and precision of our methodology, recovery experiments were performed by introducing three distinct concentrations of the fluridone standard into pristine matrices (field soil, cotton plants, and cottonseeds) devoid of the fluridone standard. Each concentration was subjected to five parallel treatments. The spiked samples were then analyzed using the methodology established in this study. Subsequently, the average recovery rates and relative standard deviations (RSDs) were computed.

### 2.7. Degradation Dynamics of Fluridone in Soil

The formula for computing the recovery rate of fluridone was provided as follows:

(2) $$R\;\left(100\%\right)=(S_{2}-S_{1})/S_{1}$$

In the formula, “*R*” represents the recovery rate of fluridone; “*S*_2_” and “*S*_1_” are the peak areas of the matrix and standard sample with the same concentration of fluridone, respectively; and “S_0_” is the peak area of the blank matrix without fluridone.

When fluridone is degraded in the soil, it typically follows a first-order kinetic law. The half-life and kinetic regression equation can be calculated using the following formulas:

(3) $$C_{t}=C_{0}e^{-kt}$$

(4) $$T_{0.5}=\ln2/k$$

In the formula, *C_t_* (mg·kg^−1^) represents the concentration of fluridone in the soil after (t) days; C_0_ (mg·kg^−1^) is the initial concentration of fluridone in the soil; k (d^−1^) is the degradation rate constant; t (d^−1^) represents the incubation time; and *T*_0.5_ (d) is the time required for fluridone to degrade to half of its initial concentration, which is also known as the degradation half-life.

### 2.8. Dietary Risk Assessment

Countries estimate the daily pesticide intake based on Formula (5) [21], while the chronic risk quotient (*RQ_c_*) is calculated using Formula (6) [22].

(5) $$NEDI=\sum [STMR_{i}\left(STMR-P_{i}\right)\times F_{i}]/bw$$

(6) $$RQ_{c}=NEDI/(ADI\times bw)\times 100\%$$

In the formula, *NEDI* stands for national estimated daily intake, with the unit of μg/(kg bw·d); *STMRi* represents supervised trials median residue, with the unit of mg·kg^−1^; *STMR-Pi* refers to supervised trial’s median residue for processed commodity, with the unit of mg·kg^−1^; *Fi* denotes the consumption of food agro-product i, with the unit of g/d; bw stands for body weight, with the unit of kg; *RQc* represents the chronic risk quotient; and *ADI* stands for acceptable daily intake, with the unit of μg/(kg bw·d).

When *RQc* ≤ 1, it indicates that the chronic risk is acceptable, and the smaller the *RQc*, the lower the risk. When *RQc* > 1, it indicates that there is an unacceptable chronic risk, and the greater the *RQc*, the higher the risk [22].

## 3. Results

### 3.1. Optimization of Testing Conditions

The results indicated that the column oven temperature, ranging from 30 °C to 40 °C, exerted minimal influence on both the peak area and retention time of the sample, thus necessitating no separate discussion. A notable trend emerged where minor variations in flow rates led to prolonged retention times for fluridone. When acetonitrile solution was employed as the organic phase, as opposed to a methanol solution, it facilitated superior separation between components and impurities, characterized by fewer stray peaks, enhanced peak areas of active ingredients, and improved peak shapes.

To enhance separation efficiency and peak morphology within an acetonitrile–water mobile phase system, a 0.1% formic acid aqueous solution was substituted for the pure water phase. The subsequent optimization of this mixture’s proportion revealed negligible effects on peak shape. To mitigate impurity interference and achieve optimal peak shape, further refinement of the mobile phase ratio and flow rate was conducted. An acetonitrile–0.1% formic acid aqueous solution system, with a volume ratio of 80:20, yielded optimal peak shapes, exhibiting minimal impurity peaks, maximum peak area, moderate retention times, and consistent results across multiple detections at a flow rate of 0.3 mL/min.

**Figure 1:**
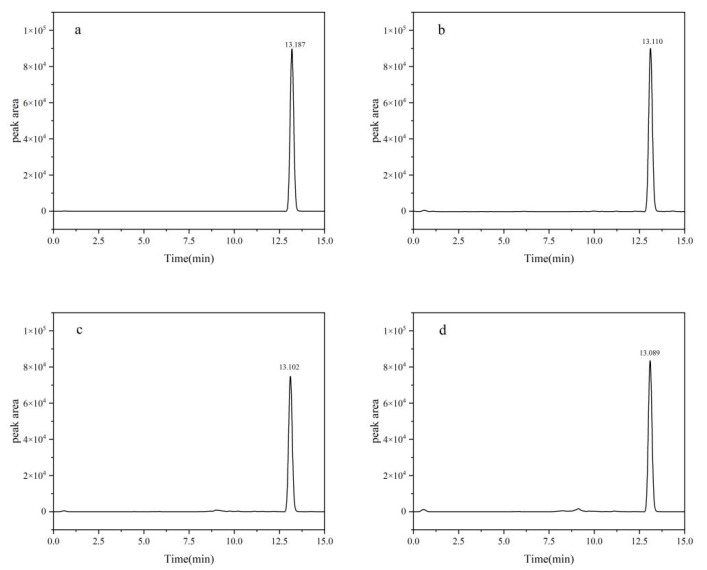
HPLC chromatograms of fluridone: (**a**) blank; (**b**) soil; (**c**) cotton plant; (**d**) cottonseed. Fluridone spiked at 10.0 mg/kg (n = 3).

The definitive chromatographic conditions were established as follows: an injection volume of 2.0 μL, a mobile phase composed of acetonitrile and 0.1% formic acid aqueous solution in an 80:20 volume ratio, a flow rate of 0.3 mL/min, and a column oven temperature precisely set at 40 °C (Figure 1).

### 3.2. Optimization of Preprocessing Methods

#### 3.2.1. Optimization of Extraction Solvents

**Figure 2:**
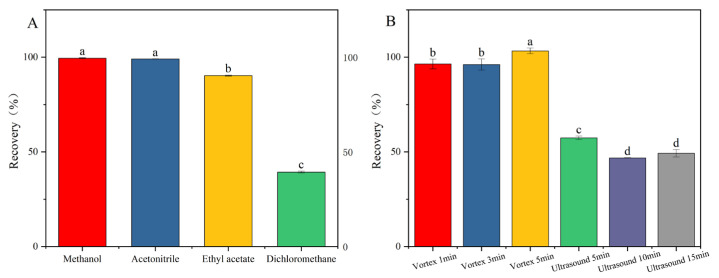
Pretreatment optimization: (**A**) extraction solvent; (**B**) extraction method. Fluridone spiked at 10.0 mg/kg (n = 3), (*p* < 0.05). Note: The lowercase letters in the figure indicate a significant difference at the 5% level.

In the analysis of pesticide residues, the selection of appropriate extraction solvents for the complete recovery of target compounds from diverse matrices constitutes a pivotal step in sample pretreatment [23,24]. Among the various solvents employed, acetonitrile, methanol, dichloromethane, and ethyl acetate are commonly utilized in pesticide residue analysis methodologies [18,19,25]. Considering the varying polarities of pesticides and their miscibility with the mobile phase, this study selected four solvents: methanol, acetonitrile, dichloromethane, and ethyl acetate. Appropriate amounts of the mixed pesticide standard solution were added to blank samples, followed by the introduction of each of the four solvents. Under identical processing conditions, addition–recovery experiments were conducted, and each group was repeated three times to ensure accuracy. The extraction efficiencies of the different solvents were then compared. As can be seen from Figure 2A, the recovery rates of the other three extraction solvents were satisfactory, except for dichloromethane. Considering the practical effectiveness and application cost, this study chose acetonitrile as the extraction solvent.

#### 3.2.2. Optimization of Extraction Methods

Under identical experimental conditions, the impact of two extraction techniques—ultrasound (5 min, 10 min, 15 min) and vortex mixing (1 min, 3 min, 5 min)—on the recovery rate of fluridone was investigated. Figure 2B demonstrates that vortex mixing extraction yields a significantly higher recovery rate compared to ultrasound extraction. This disparity can be attributed to the inherent characteristics of the two methods. Vortex mixing facilitates the rapid and thorough mixing of the sample, ensuring complete dissolution of fluridone. Conversely, ultrasound extraction may not achieve the complete dissolution of fluridone within the sample, leading to a lower recovery rate. When vortex mixing was applied for 5 min, the recovery rate peaked, thus fulfilling the extraction requirements. Therefore, in this study, vortex mixing for 5 min was adopted as the optimal method for extracting fluridone from the sample.

#### 3.2.3. Optimization of Salt

**Figure 3:**
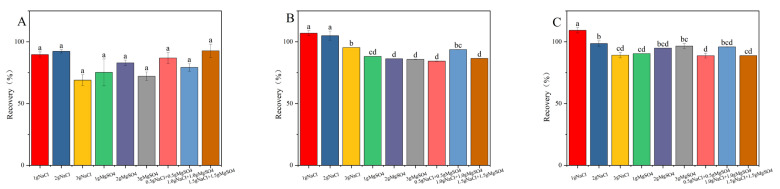
The effects of different salt contents on the recovery rate of fluridone in the (**A**) soil, (**B**) cotton plant, and (**C**) cottonseeds. Fluridone spiked at 10.0 mg/kg (n = 3). Note: The lowercase letters in the figure indicate a significant difference at the 5% level.

Sodium chloride serves as a potent facilitator in liquid–liquid extraction processes, inducing a salting-out effect that enhances the recovery efficiency of target compounds. Furthermore, anhydrous magnesium sulfate exhibited a remarkable capacity for water binding, thereby substantially reducing the aqueous phase and promoting the preferential distribution of target compounds within the organic layer. The synergistic application of sodium chloride and anhydrous magnesium sulfate not only allows for the precise modulation of the extraction medium’s polarity, but also optimizes the peak characteristics of the targeted analytes [19,26,27]. Under consistent experimental parameters, the impact of varying NaCl (1.0 g, 2.0 g, 3.0 g) and MgSO_4_ (1.0 g, 2.0 g, 3.0 g) concentrations, as well as combined treatments of 0.5 g NaCl + 0.5 g MgSO_4_, 1.0 g NaCl + 1.0 g MgSO_4_, and 1.5 g NaCl + 1.5 g MgSO_4_ on the recovery rate of fluridone, was meticulously examined. As illustrated in Figure 3, the addition of 2 g of NaCl resulted in the recovery rate of fluridone in soil, cotton plants, and cottonseeds approaching 100% and enhanced its stability, thus meeting our experimental objectives. Consequently, we opted for the inclusion of 2 g NaCl in the fluridone extraction process from the samples.

#### 3.2.4. Optimization of Purification Conditions

During the extraction of target compounds from the cotton field environmental samples using acetonitrile, a substantial quantity of matrix compound was concurrently extracted. These co-extracted substances have the potential to interfere with target analytes, resulting in significant matrix effects. Post-extraction purification is therefore indispensable.

**Figure 4:**
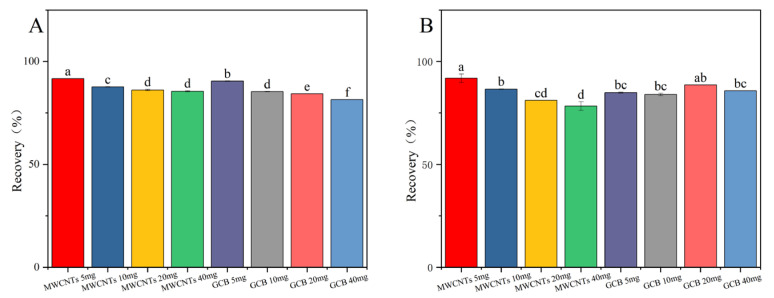
The effects of different MWCNTs and GCB contents on the recovery rate of fluridone in (**A**) cotton plant and (**B**) cottonseeds. Fluridone spiked at 10.0 mg/kg (n = 3), (*p* < 0.05). Note: The lowercase letters in the figure indicate a significant difference at the 5% level.

Graphitized Carbon Black (GCB) is a weakly polar or nonpolar adsorbent. It is mainly used for the removal of compounds through hydrophobic interactions. Examples of such compounds include carotenoids and carotenoid-like substances [27,28]. Multi-walled carbon nanotubes (MWCNTs), due to their extensive surface area and distinctive structural characteristics, possess remarkable adsorption capabilities. They can effectively eliminate interfering matrix substances while using a minimal amount of adsorbent [29,30]. The results presented in Figure 4 demonstrate that in the cotton plants and cottonseed samples, the recovery rate of fluridone was maximized when 5.0 mg of MWCNTs was employed. Nevertheless, as the quantity of MWCNTs increases, the recovery rate of fluridone shows a decline. MWCNTs display superior performance in removing pigments from the cotton plant and cottonseed matrix, yielding clearer treated solutions. Even with a relatively small amount of MWCNTs, satisfactory recovery rates were achieved. Consequently, MWCNTs (5.0 mg) were selected as the purification agents for this experiment.

### 3.3. Method Validation

#### 3.3.1. The Linearity, Limit of Detection (LOD), Limit of Quantitation (LOQ), and Matrix Effect of the Method

The standard curves for matrix-matched and pure solvent samples were constructed following the method described in Section 2.2. Subsequently, the matrix effect was calculated. The linear regression equation, limit of detection (LOD), limit of quantitation (LOQ), and matrix effect of fluridone in various samples are presented in Table 2. The results demonstrated a strong linear relationship (R^2^ > 0.99) between the concentration of fluridone and the peak area in field soil, plant, and cottonseed samples within the concentration range of 0.01 to 10 mg/L.

LOD and LOQ are crucial validation parameters for assessing the sensitivity of analytical methods. The LOD of fluridone in the samples ranged from 0.00090 to 0.00108 mg·kg^−1^, and the LOQ ranged from 0.0030 to 0.0033 mg·kg^−1^. According to GB2763.1-2022, the LOD is lower than the maximum residue limit standard for pesticides in food, which is 0.02 mg·kg^−1^. Moreover, the LOD and LOQ values obtained by this method are lower than those reported by Luo, Juan et al. in their determination of isocyanamide residues in cabbage and soil samples using high-performance liquid chromatography (HPLC) [19].

#### 3.3.2. The Accuracy and Precision of This Method

As presented in Table 3, the average daily recoveries of fluridone in field soil samples spanned from 89.08% to 95.07%. The relative standard deviation of repeatability (RSDr) values fell within the range of 2.06% to 4.57%. Regarding cottonseed samples, the average daily recoveries were in the range of 85.81% to 90.72%, with RSDr values ranging from 0.95% to 2.76%. For cotton plant samples, the daily recoveries were between 85.08% and 89.89%, and the RSDr values were from 0.35% to 0.78%. The daily precision was evaluated by analyzing spiked samples, and the relative standard deviation of repeatability (RSDR) values were found to be in the range of 0.47% to 3.81%. The recoveries of fluridone in all three types of samples were within the 70–120% range, and the relative standard deviation (RSD) was less than or equal to 20%. These results comply with the requirements specified in the Chinese Guidelines for Crop Pesticide Residue Testing (GBNY/T 788-2018). Thus, this method is suitable for the detection of fluridone and demonstrates good detection accuracy.

### 3.4. Degradation Dynamics of Fluridone in Cotton Field Soil

Pesticides sprayed onto the soil undergo various degradation processes. Pesticide loss is influenced by the pesticide’s physicochemical properties, soil characteristics, climatic conditions, and pesticide application methods [26,31]. Table 4 presents the kinetic equation parameters for the degradation of fluridone in cotton fields during the period 2023–2024.

**Figure 5:**
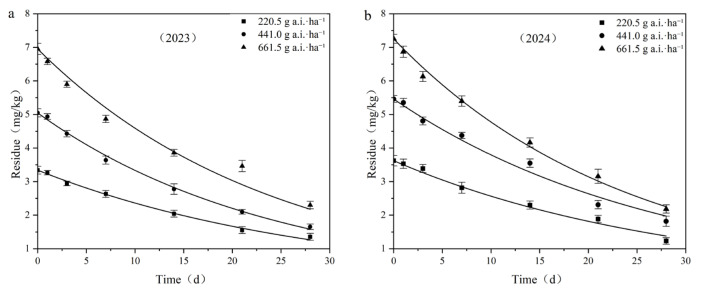
Degradation dynamics curve of fluridone in cotton field soil: (**a**) 2023; (**b**) 2024.

Taking the sampling time after application as the abscissa and the residual concentration of fluridone in the samples as the ordinate, dynamic curves of the residual degradation of different fluridone concentrations in field soil were plotted, as depicted in Figure 5. The residues of fluridone measured 2 h after application were regarded as the initial deposition.

When the application doses were 220.5 g a.i.·ha^−1^, 441.0 g a.i.·ha^−1^, and 661.5 g a.i.·ha^−1^, the initial deposition in the field ranged from 3.331 mg·kg^−1^ to 7.247 mg·kg^−1^, with degradation half-lives ranging from 16.503 days to 21.004 days, respectively.

The results of the fluridone residual dissipation tests indicated that the initial deposition of fluridone increased with the increase in the applied dose, while the half-life decreased as the application dose increased. It is reasonable to assume that various environmental factors, such as soil microorganisms, precipitation levels, temperature, and light intensity, may have an impact on the degradation process of fluridone.

### 3.5. Final Residues of Fluridone in Cotton Field Soil, Cotton Plants, and Cottonseeds

Measuring the residue levels at harvest is of crucial significance for assessing the safety of using fluridone to control weeds in cotton fields. In Table 5, the findings revealed that during the period 2023–2024, the residue levels of fluridone in the cotton field soil ranged from 0.032 mg/kg to 0.155 mg/kg. In cotton plants, these levels ranged from 0.215 mg/kg to 0.412 mg/kg. Moreover, in cottonseeds, the residue levels were below the limit of quantitation (LOQ). These results suggest that the final residue levels of fluridone in cotton field soil were relatively elevated, presenting a higher risk of environmental pollution. Additionally, the relatively high residue levels in cotton plants imply a potential risk if the plants are used as forage. Nevertheless, it is notable that the final residue levels in cottonseeds are below the LOQ.

### 3.6. Dietary Risk Assessment

Final residue testing not only examines the final residue levels of pesticides applied to crops, but also assesses the potential risks associated with dietary intake [17,25]. Dietary risk assessment is a crucial element in the evaluation of agricultural product quality and safety and serves as one of the measures to gauge the safety of agricultural products.

Cottonseed, an important byproduct of cotton production, is a significant feed resource. This is attributed to its rich nutritional composition and high yield. Cottonseed is abundant in various nutrients, including protein, fat, and fiber. These nutrients can supply the essential energy and nutrition for animals, and also enhance the production performance and health status of livestock and poultry [32].

The maximum residue limit for pesticides in food stipulates that the acceptable daily intake (ADI) of fluridone is 0.15 mg·kg^−1^·bw. In this experiment, the residues in cottonseed were below the limit of quantification (LOQ). When fluridone was applied at rates of 220.5 g a.i.·ha^−1^, 441.0 g a.i.·ha^−1^, and 661.5 g a.i.·ha^−1^, no significant health risks were posed to Chinese consumers through the cottonseed oil extracted from cottonseed.

## 4. Conclusions

In this study, a relatively straightforward and rapid QuEChERS pretreatment coupled with the HPLC method was developed to determine fluridone residues in field soil, cotton plants, and cottonseeds. The validation results demonstrated that the proposed method exhibited excellent linear correlation, accuracy, precision, LOD, and LOQ, thereby meeting the requirements for pesticide residue analysis.

The degradation of fluridone in the field adhered to first-order kinetics. Notably, the degradation half-life of fluridone in the soil was relatively short. A dietary risk assessment revealed that the residue levels of fluridone in harvested cottonseeds did not pose a significant health risk to Chinese consumers.

At present, certain countries and regions have not yet established maximum residue limits (MRLs) for fluridone, and the understanding of its residues in food and the environment remains limited. Consequently, this study holds great significance for the rational application of fluridone. It offers a reference for the establishment of MRLs and the safe utilization of fluridone. Moreover, it provides a foundation for subsequent investigations of the environmental behavior of soil.

However, it should be noted that this study only covered a limited number of sampling points and a relatively short time period. This limitation could potentially restrict a comprehensive understanding of the degradation dynamics of fluridone. A larger number of sampling points and long-term monitoring are recommended to obtain more accurate and comprehensive data.

In addition, a large number of studies have shown that soil texture, soil microorganisms, temperature, soil moisture content, etc., will affect the digestion of pesticides in soil [33]. This study only focused on certain soil types, thus failing to encompass all possible soil types. Consequently, future research should take into account the impact of different soil types on the degradation dynamics and residues of fluridone.

Furthermore, although this study identified the degradation half-life of fluridone in soil, the specific degradation mechanism remains inadequately understood. Further research on the degradation mechanism will enhance our understanding of the behavior of fluridone in soil and provide a scientific basis for its management.

## Figures and Tables

**Table 1:** Three levels of addition concentrations for the addition recovery test.

Sample Type	Low	Middle	High
Soil	0.1	0.5	1
Cotton plant	0.1	0.5	1
Cottonseed	0.1	0.5	1

**Table 2:** Linear regression equation, limit of detection (LOD), limit of quantitation (LOQ), and matrix effect of fluridone in different matrices.

Matrix	Regression Equation	R^2^	LOD (mg·kg^−1^)	LOQ (mg·kg^−1^)	Matrix Effects (%)
Blank	y = 14,465.8x + 6524.04	0.9999159	/	/	/
Soil	y = 14,536.7x + 4981.41	0.9998105	0.00090	0.0030	0.49
Plant	y = 15,606.5x + 6476.5	0.9921023	0.00108	0.0036	7.89
Cottonseed	y = 15,868.4x + 872.01	0.9996730	0.00099	0.0033	8.84

**Table 3:** Daily average recoveries and relative standard deviations of fluridone in cotton field soil, cotton plant, and cottonseed samples. Intraday RSDr refers to the relative standard deviation of repeatability (n = 5); interday RSDr refers to the relative standard deviation of reproducibility (n = 15).

Matrix	Spiked-Level	Intra-Day (n = 5)						Inter-Day (n = 15)	
		Day1	RSDr	Day2	RSDr	Day3	RSDr		RSDr
	(mg·kg^−1^)	Average Recoveries		Average Recoveries		Average Recoveries		Average Recoveries	
		(%)	(%)	(%)	(%)	(%)	(%)	(%)	(%)
Soil	0.1	93.55	2.70	92.16	4.46	89.08	2.59	91.60	3.25
	0.5	94.50	4.57	90.35	3.57	89.14	3.29	91.33	3.81
	1	95.07	2.82	91.22	2.06	89.75	3.09	92.01	2.66
Cottonseed	0.1	87.40	2.76	87.49	1.49	85.81	1.24	86.90	1.83
	0.5	89.09	1.37	87.25	1.26	88.67	0.95	88.34	1.19
	1	90.72	2.18	89.06	0.61	88.18	0.98	89.32	1.26
Plant	0.1	86.80	0.63	85.72	0.44	85.08	0.35	85.87	0.47
	0.5	88.01	0.34	86.90	0.73	86.29	0.44	87.07	0.50
	1	89.89	0.78	88.00	0.46	86.69	0.46	88.19	0.56

**Table 4:** Parameters of the residual degradation dynamics equation for fluridone in cotton field soil.

Year	Dosage (g a.i.·ha^−1^)	Equation	Correlation Index (R^2^)	k (Day^−1^)	Half-Life (Days)
	220.5	$y=3.331e^{-0.033t}$	0.996	0.033	21.004
2023	441.0	$y=5.065e^{-0.042t}$	0.997	0.042	16.503
	661.5	$y=6.944e^{-0.038t}$	0.981	0.038	18.241
	220.5	$y=3.647e^{-0.035t}$	0.986	0.035	19.804
2024	441.0	$y=5.462e^{-0.037t}$	0.987	0.037	18.734
	661.5	$y=7.247e^{-0.041t}$	0.991	0.041	16.906

**Table 5:** Final residues of fluridone in cotton field soil, cotton plants, and cottonseeds.

Year	Materials	Dosage (g a.i.·ha^−1^)	Final Residue (mg·kg^−1^)
		220.5	0.067
	Soil	441.0	0.077
		661.5	0.155
		220.5	0.234
2023	Plant	441.0	0.322
		661.5	0.412
		220.5	<LOQ ^a^
	Cottonseed	441.0	<LOQ ^a^
		661.5	<LOQ ^a^
		220.5	0.032
	Soil	441.0	0.075
		661.5	0.134
		220.5	0.215
2024	Plant	441.0	0.341
		661.5	0.402
		220.5	<LOQ ^a^
	Cottonseed	441.0	<LOQ ^a^
		661.5	<LOQ ^a^

Note: ^a^ <LOQ represents below the limit of quantification.

## Data Availability

The data presented in this study are available on request from the corresponding authors.

## Supplementary Materials

The following supporting information can be downloaded at: https://www.mdpi.com/article/10.3390/toxics13070526/s1, Table S1: Physicochemical properties of the tested soils.
