# Supplementary material for: Analysis of the Digestion Dynamics and Dietary Risk Assessment of Fluridone in Cotton Fields via QuEChERS Coupled with HPLC
Source: Toxics. 2025 Jun 23;13(7):526. doi: 10.3390/toxics13070526 (PMC12299286; doi:10.3390/toxics13070526)
Supplement: Supplementary file 1 [file toxics-13-00526-s001.zip › toxics-3682792-supplementary.pdf]

Supplementary Material

Analysis of the Digestion Dynamics and Dietary Risk As sessment of Fluridone in Cotton Fields via QuEChERS Coupled with HPLC

Table S1: Physicochemical properties of the tested soils

year	pH	EC	AK	AP	AN	SOM
2023	7.77±0.10	374.00±7.21	456.00±3.07	27.16±0.27	50.23±16.79	38.42±0.57
2024	7.92±0.07	436.10±10.52	319.23±2.42	19.65±0.16	33.73±6.57	34.27±0.46

Note: EC- electrical conductivity; AK- available k; AP- available P; AN- alkali-hydrolyzable nitrogen; SOM- Soil organic matter.
